# *Lv*YY1 Activates WSSV *ie1* Promoter for Enhanced Vaccine Production and Efficacy

**DOI:** 10.3390/vaccines8030510

**Published:** 2020-09-08

**Authors:** Li-Na Tao, Ze-Hui Liu, Hui-Ling Xu, Ying Lu, Min Liao, Fang He

**Affiliations:** 1 Institute of Preventive Veterinary Medicine, College of Animal Sciences, Zhejiang University, Hangzhou 310058, China; 21817049@zju.edu.cn (L.-N.T.); zehuiliu@zju.edu.cn (Z.-H.L.); 11917012@zju.edu.cn (H.-L.X.); 21917097@zju.edu.cn (Y.L.); 2 Key Laboratory of Animal Virology of Ministry of Agriculture, Zhejiang University, Hangzhou 310058, China; liaomin4545@zju.edu.cn

**Keywords:** BEVS, WSSV *ie1* promoter, *Lv*YY1, transcription enhancer, CSFV E2, AIVs H5

## Abstract

The baculovirus expression vector system (BEVS) has been used as a preferred platform for the production of recombinant protein complexes and efficacious vaccines. However, limited protein yield hinders the application of BEVS. It is well accepted that transcription enhancers are capable of increasing translational efficiency of mRNAs, thereby achieving better protein production. In this study, the ability of *Lv*YY1 as a transcription enhancer was assessed. *Lv*YY1 could interact with the WSSV *ie1* promoter via binding to special DNA sites in BEVS. The effects of *Lv*YY1 on protein expression mediated by WSSV *ie1* promoter of BEVS was investigated using eGFP as a reporter gene. Enhanced eGFP expression was observed in Sf-9 cells with *Lv*YY1. On this basis, a modified vector combining *ie1* promoter and *Lv*YY1 was developed to express either secreting CSFV E2 or baculovirus surface displayed H5 HA of AIVs. Compared to control groups without *Lv*YY1, E2 protein yield increases to 1.6-fold, while H5 production improves as revealed by an upregulated hemagglutination titer of 8-fold at least. Moreover, with *Lv*YY1, H5 displaying baculovirus driven by WSSV *ie1* promoter (BV-*Lv*YY1-*ie1*-HA) sustains the transduction activity in CEF cells. In chicken, BV-*Lv*YY1-*ie1*-HA elicits a robust immune response against H5 AIVs in the absence of adjuvant, as indicated by specific antibody and cytokine responses. The findings suggest its potential function as both a vectored and subunit vaccine. These results demonstrate that the coexpression with *Lv*YY1 serves as a promising strategy to extensively improve the efficiency of BEVS for efficacious vaccine production.

## 1. Introduction

The baculovirus expression vector system (BEVS) has been used as a powerful tool for vaccine development [1,2]. In contrast to other vectors, BEVS incurs much less biosafety concerns for human and animal applications. In addition, there are other advantages of the baculovirus expression system, including expression of large proteins which are usually correctly folded and biologically active, efficient cleavage of signal peptides, and processing of the protein. This greatly facilitates complex protein production. Thus, BEVS has become the most widely used system in the production of recombinant protein.

BEVS allows the production of different vaccine types, including secreting protein and viral surface displayed antigen. Glycoprotein E2 of classical swine fever virus (CSFV) is widely used as a secreting subunit vaccine candidate against classical swine fever (CSF), which causes huge economic losses worldwide in the pig industry [3,4]. Recombinant E2 of BEVS can efficiently secret to culture supernatant [5]. Thus, E2 is widely used as a model to design subunit vaccines, especially secretion antigen [6,7,8,9].

H5 subtype avian influenza poses a serious threat to public health, as well as to poultry industries [10,11,12]. There are three main types of influenza vaccines based on BEVS: surface displayed vaccines, recombinant HA vaccines, and virus-like particle vaccines, among which *ie1* promoter-driven transcription has been applied to express surface displayed HA [13,14]. Furthermore, surface displayed HA sustains the correct biological activity as original AIV HA, leading to the efficacious immunogenicity when used as vaccines.

In most cases, the high expression levels of foreign protein rely on baculovirus transcription in infected cells in late phases with the viral polyhedrin (*polh*) and p10 promoters [15]. Although the viral *polh* and p10 promoters mediate better protein expression [16], it is greatly disturbed with cell apoptosis in the late phase of infection. In light of this limitation, considerable research efforts have been channeled into increasing the productivity of BEVS. For example, a number of novel sequences (vankyrins) from an insect virus (polydnavirus) have been reported [17], which significantly delay death and lysis of baculovirus infected cells while enhancing recombinant protein production. Moreover, some genes have been shown to prolong the life of baculovirus-infected cell cultures by inhibiting the induction of apoptosis, such as bacl-2 [18] and baculovirus P35 protein [19]. In addition, the viral promoter modification is also a good strategy to prolong infection, such as the employment of white spot syndrome virus (WSSV) immediate-early one (*ie1*) promoter in baculovirus [20]. With WSSV *ie1* promoter, protein expression starts early during infection and continues through the late phase, leading to improvement in the protein yield.

WSSV is a DNA virus in shrimp with one of largest genome of 300 kbp [21]. It was initially classified as belonging to the baculovirus family according to the similarity in genome and the tropism in invertebrates, although it has now been categorized to its own family. WSSV *ie1* promoter has been proven to be active in BEVS and to be even stronger than cytomegalovirus immediate-early (CMV) promoter [22] and *polh* promoter at the early phase [23]. Hence, it has been widely used to improve the expression of foreign proteins in BEVS for human and animal vaccine development, such as influenza H1 [24] and WSSV VP28 [25]. Meanwhile, recombinant baculoviruses with WSSV *ie1* promoter induced stronger immune responses than those with CMV promoter [26]. For example, in chicken, baculovirus containing WSSV *ie1* promoter elicited more significant HA-specific antibodies and hemagglutination inhibition (HI) titers compared to those with CMV promoter [22]. It has been reported that recombinant baculoviruses driven by WSSV *ie1* promoter (BV-*ie1*-gp64-VP1) or *polh* promoter (BV-*polh*-gp64-VP1) successfully surface-displayed chimeric major immunogenic capsid protein (VP1) of human enterovirus 71 (EV71). BV-*ie1*-gp64-VP1-immunized mice were found to induce higher neutralization antibody titers than BV-*polh*-gp64-VP1 [27]. Furthermore, WSSV *ie1* promoter is active in different cell types, including most vertebrate cells, suggesting the promising application as a consensus vectored vaccine.

In order to further improve the potential of WSSV *ie1* promoter for enhanced vaccine production, efforts are being made to activate or engineer the promoter with different strategies. Enhanced protein secretion may be obtained by coupling to enhancer elements, such as homologous region 1 and the delayed-early 39K gene [28,29]. Therefore, translation enhancer, as a translation regulatory element, can improve protein expression by enhancing the efficiency of mRNA translation to a certain extent. It is an alternative strategy to improve the productivity of the baculovirus expression system.

Yin Yang1 (YY1) regulates transcription and has four highly conserved Cys2-His2 zinc finger DNA-binding domains, which can bind to a consensus binding sequence [30,31,32]. It was reported that *Lv*YY1 from shrimp (*Litopenaeus vannamei*, *Lv*YY1) activates WSSV *ie1* expression by a *Lv*YY1-binding site and TATA box in *ie1* promoter [30], but whether *Lv*YY1 would function in BEVS is still unknown. Thus, for the first time, in the present study, it was investigated whether transcription enhancer, together with the certain promoter, would enhance the recombinant protein expression and improve vaccine efficacy accordingly.

In this study, we aimed to improve the productivity of the baculovirus expression system by coupling enhancer element (*Lv*YY1) and verify the product efficacy in target animals. *Lv*YY1 was expressed in baculovirus and confirmed to enhance protein expression via the binding to *ie1* promoter. To explore the effects of *Lv*YY1 on promoter-mediated gene expression, a series of baculovirus expression vectors were developed and screened. Using secreting CSFV E2 [5] and surface displayed AIV HA [33], as models, the modified vector was tested as an alternative strategy to improve the production of novel vaccines in Sf-9 cells. The efficacy of the enhanced expression of HA with *Lv*YY1 was evaluated in chicken against AIV.

## 2. Materials and Methods

### 2.1. Viruses and Cells

Inactivated influenza virus H5N1 (A/Pika/Qinghai/BI/2007) was a gift from Key Laboratory of Animal Virology of Ministry of Agriculture, Zhejiang University. Virus was prepared as described before [34]. *Spodoptera frugiperda* (Sf-9) cells were maintained in Sf900 Ⅲ SFM (Invitrogen, Carlsbad, CA, USA) at 27.5 °C for recombinant baculovirus production. CEF cells were prepared from 11-day-old specific pathogen-free (SPF) chicken egg embryos by standard procedures and cultured in DMEM (ThermoFisher, Waltham, MA, USA) containing 10% fetal bovine serum (FBS, ThermoFisher, Waltham, MA, USA).

### 2.2. Construction of Baculoviral Expression Vectors Containing LvYY1

**Figure 1:**
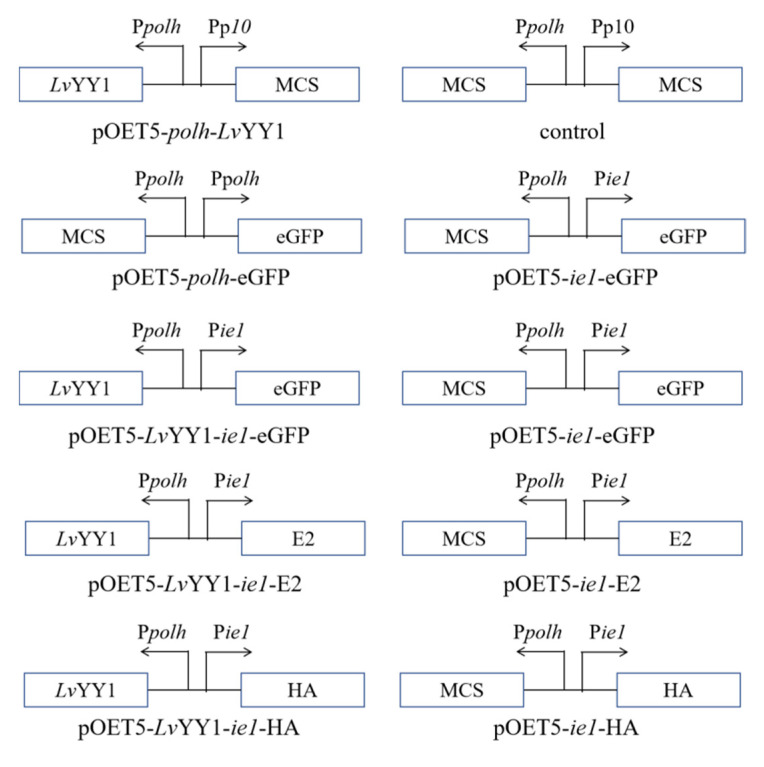
Schematic diagram of donor vectors of recombinant baculoviruses. Arrows indicated the direction of the promoters to drive the protein expression. *ie1*: WSSV immediate-early one promoter, *Lv*YY1: *Litopenaeus Vannamei* Yin Yang 1, MCS: multiple cloning sites, *polh*: AcMNPV polyhedrin promoter, p10: AcMNPV p10 promoter, eGFP: Enhanced Green Fluorescent Protein, E2: Glycoprotein E2 of CSFV, HA: Hemagglutinin of AIVs.

Transcription enhancer *Lv*YY1 (GenBank accession no.KT820172.1), with a Flag tag at its C-terminus, was synthesized and subcloned into baculoviral expression vectors to generate pOET5-*polh*-*Lv*YY1. Two plasmids, pOET5-*ie1*-eGFP and pOET5-*Lv*YY1-*ie1*-eGFP, were constructed using standard procedures, as illustrate in Figure 1. In short, *ie1* and eGFP genes were amplified from pFast HTB and pFBDM plasmid using primer pairs ie1-F/R and eGFP-F/R, respectively. Amplied gene fragments were used to generate ie1-eGFP via overlapping PCR. PCR products were purified with a Cycle Pure Kit (Omega, Norcross, Georgia, USA) and cloned into pOET5 Vector. At least three positive clones were sequenced using the Sanger sequencing approach. E2/HA expressing vectors were generated by replacing eGFP. The resultant plasmids were verified by DNA sequencing. All primers used in this study are listed in Table 1.

### 2.3. Generation and Titration of Recombinant Baculovirus

Correct plasmids were co-transfected with bacmid using Cellfectin II Reagent (Invitrogen, Carlsbad, CA, USA) as directed by the manufacturer to generate recombinant baculoviruses. P1 viruses were collected after incubation of transfected cells at 27.5 °C for 7 days. High titer virus stocks were made by passages in Sf-9 cells. The titer of baculovirus stocks was tested by end-point dilution assay, which was performed as previously described [35]. Then every virus stock titer was calculated according to Reed–Muench method.

### 2.4. Immunofluorescence Assay (IFA)

Sf-9 cells were seeded at a concentration of 2 × 10^5^ cells/well into 6-well plates and infected with virus at MOI of 0.1. At 48 h post infection, Sf-9 cell monolayers were washed 3 times with PBS and fixed for 20 min with precooled 80% acetone. Fixed Sf-9 cells were incubated with anti-Flag monoclonal antibody (TransGen Biotech, Beijing, China), followed by TRITC-coupled goat anti-mouse IgG (Thermo Scientific, MA, USA). Then Sf-9 cell monolayers were labeled nuclear DNA using 4′,6′-diamidino-2-phenylindole dihydrochloride (DAPI, Beyotime, Shanghai, China). *Lv*YY1 proteins were viewed with an inverted fluorescence microscope (Olympus, Tokyo, Japan) and then merged by image J (National Institutes of Health, Bethesda, Maryland, USA).

### 2.5. Western Blot

A quantity of 1.5 × 10^6^ Sf-9 cells in 2 mL of medium were seeded in each well of 6-well plates and infected with virus at an MOI of 0.1. Recombinant baculovirus samples were collected and processed in the same volume of different test groups at 12, 24, 36, and 48 h, respectively. Protein samples prepared equally were loaded and separated by SDS-12% polyacrylamide gel electrophoresis. Target proteins were transferred to polyvinylidene fluoride (PVDF) membranes (Merck Millipore, Darmstadt, Germany) and blocked for 1.5 h at 37 °C with 5% (wt/vol) nonfat milk in TBST buffer containing Tween 20. Membranes were incubated with anti-Flag monoclonal antibody (TransGen Biotech, Beijing, China) or GP64 monoclonal antibody (Abcam, Cambridge, UK) overnight at 4 °C. After rinsing with TBST, membranes were incubated with HRP-conjugated goat anti-mouse IgG (Sungene, Shanghai, China) at 37 °C for 1 h and further washed with TBST 3 times; immunoreactive bands were visualized by Super Signal West Pico/Femto Chemiluminescent Substrate (Thermo Scientific, MA, USA), and images were captured using a Gel 3100 chemiluminescent imaging system (Sage Creation Science, Beijing, China).

### 2.6. Flow Cytometry

Sf-9 cells infected with recombinant baculovirus were collected at 12, 24, 36, and 48 h, respectively. The cells were washed twice with PBS, collected by centrifugation (5 min at 1000 rpm), and the concentration was resuspended in PBS for flow cytometry analysis. Then 1 × 10^4^ live Sf-9 cells per sample were used for detection of fluorescence number and fluorescence intensity. All flow cytometric experiments were performed with a BD FACSCalibur flow cytometer (BD Biosciences, New York, NY, USA). Data were analyzed using FlowJo software (version 10; TreeStar, San Carlos, CA, USA).

### 2.7. Gel Shift Assays

*Lv*YY1 protein was produced by using pET-28a vector that carried an N-terminal His tag and purified by Ni-nitrilotriacetic acid (NTA) agarose column chromatography (Sangon Biotech, Shanghai, China). Nuclear proteins from Sf-9 cells infected with *Lv*YY1 expressing baculovirus were extracted by using NE-PER nuclear and cytoplasmic extraction reagents (Thermo Scientific, MA, USA) according to the manufacturer’s instructions. Then proteins were quantified using an enhanced BCA protein assay kit (Beyotime, Shanghai, China). WSSV *ie1* promoter DNA and the mutant *ie1* DNA fragment were prepared by PCR. For supershift assays, 200 ng *ie1* promoter DNA and the mutant *ie1* DNA fragment were mixed with different concentrations (1–5 ug) of purified *Lv*YY1 protein or nucleoprotein in binding buffer. Mixtures were incubated at 37 ℃ for 30 min and chilled on ice for 30 min. The sample was mixed with 6× loading buffer (CWBIO, Beijing, China), and subjected to electrophoresis in 1% nucleic acid gel. Images were obtained using a JS-680B automatic gel imaging analysis system (P&Q S&T, Shanghai, China).

### 2.8. Hemagglutination Assays

A hemagglutination assay was performed in V-bottom micro-titer plates using an HA antigen. Samples were 2-fold diluted in PBS. Wells that contained no antigen at all served as negative controls. Results were read after 30 min of incubation with 1% chicken red blood cells (RBCs) at room temperature.

### 2.9. Transduction and Quantitative PCR (qPCR)

Transduction efficiency of CEF cells with recombinant baculovirus vaccines was also a critical checkpoint. A third passage (P3) of baculoviruses (BV-*Lv*YY1-*ie1*-HA) was transduced with CEF cells using an MOI of 100 for 12 h. The culture were replaced with fresh DMEM (Thermo Scientific, MA, USA) and incubated for 48 h before being fixed for immunofluorescence assay (IFA) analysis as described above. BV-*Lv*YY1-*ie1*-HA-transduced cells were washed with precooled PBS three times. Total RNAs were extracted using an RNAsimple Total RNA Kit (Tiangen, Beijing China) and were reverse transcribed into cDNA using HiScript Q RT SuperMix for qPCR (Vazyme Biotech, Piscataway, NJ, USA). cDNA was used as the template for qPCR by using the primers (Table 1) targeting the HA5 genomic region.

### 2.10. Animal Study

Fourteen-day-old SPF chickens were randomly divided into six groups (*n* = 5). Chickens were intramuscularly injected with different hemagglutination titers (2^8^, 2^6^, 2^4^) of 200 μL live baculoviruses containing HA antigen without adjuvant (BV-*ie1*-HA, BV-*Lv*YY1-*ie1*-HA) and commercial inactivated vaccine (QYH, Beijing, China) every 14 days. PBS was used as an unvaccinated control. The sera were collected two weeks after each vaccination for evaluation. All experiments involving live viruses and animals were performed in biosafety level 2 animal facilities in accordance with the institutional biosafety manual.

### 2.11. Serological Assays

The commercially available hemagglutinin ELISA kit (Jining shiye, Shanghai, China) was used to detect quantitatively hemagglutinin antibodies in chickens. Briefly, all reagents and ELISA strips were returned to room temperature before starting the assay procedure. Then 50 uL of serum and standard samples were added to the wells, followed by 100 uL of HRP-conjugated HA 5 subtype antibody. The reaction well was sealed with an adhesive strip and incubated at 37 °C for 1 h. The incubation mixture was discarded and washed 5 times by 1× wash solution. Substrates A and B were added to wells and incubated at 37 °C for 15 min. Finally 50 uL of stop solution were added, and the OD value at 450 nm was measured. The levels of interleukin (IL)-4 and interferon-γ (IFN-γ) were determined by ELISA (Jining shiye, Shanghai, China). The ELISA test was performed following the manufacturer’s manual, and the concentrations of IgG, IL-4, and IFN-γ were calculated using standard linear regression curves.

### 2.12. Hemagglutination Inhibition (HI)

Sera were further tested in HI assays [36]. In brief, all serum samples were firstly treated with receptor-destroying enzyme (RDE). Twofold serially diluted serum in PBS was incubated with 4 hemagglutination (HA) units of the H5N1 viruses (A/Pika/Qinghai/BI/2007), at 37 °C for 20–30 min. HI tests were then carried out in microtiter plates with 1% suspension of chicken RBCs.

### 2.13. Statistical Analysis

All results in figures were presented, where appropriate, as mean ± standard deviation (SD) from three independent experiments and analyzed using Student’s *t*-test. Differences were considered significant with *p* values of <0.05 and highly significant with *p* values of <0.01.

### 2.14. Ethical Approval and Informed Consent

Animal experiments were approved by the Animal Welfare and Ethics Committee at Laboratory animal center of Zhejiang University (approval number 13024). All experiments were performed in accordance with relevant guidelines and regulations.

## 3. Results

### 3.1. LvYY1 Expression and Interaction with WSSV ie1 Promoter in Baculovirus

**Figure 2:**
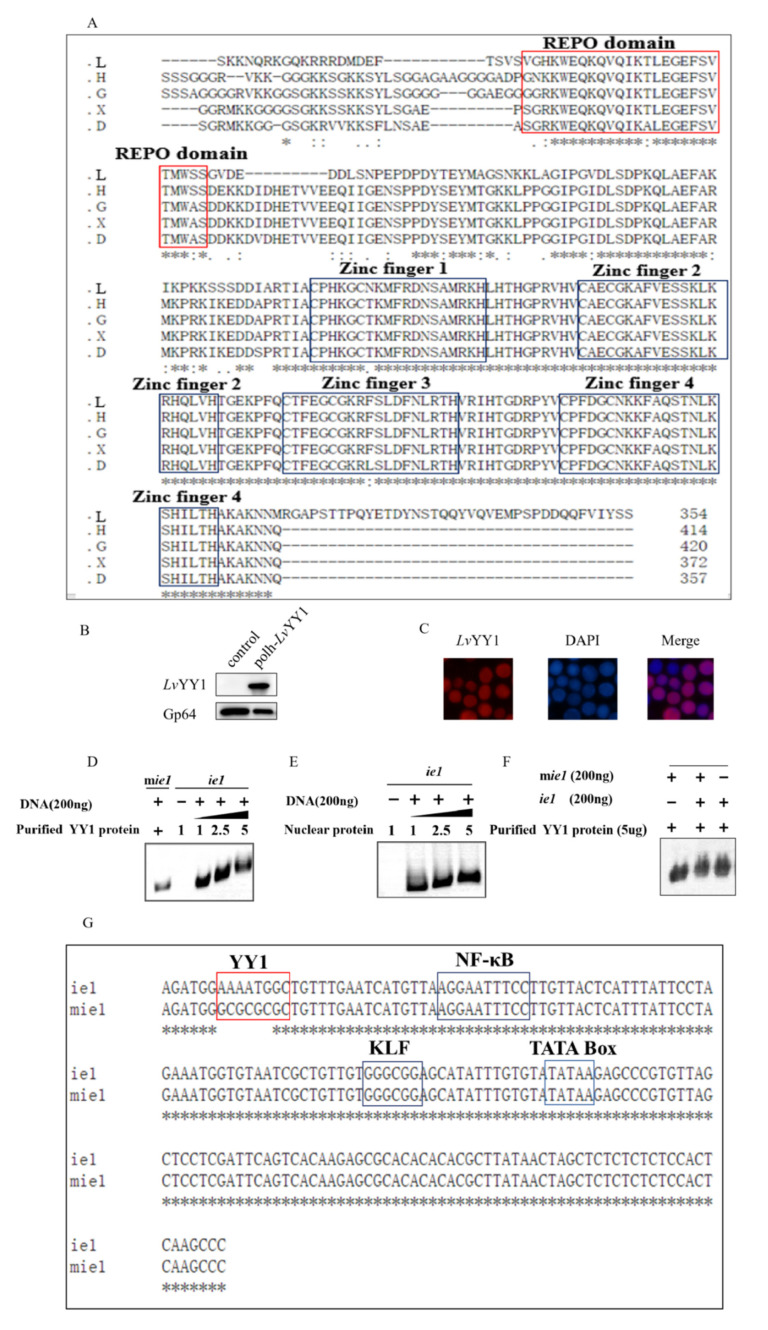
*Lv*YY1 expressed in Sf-9 cells and bound to WSSV *ie1* promoter. (**A**) *Lv*YY1 (GenBank accession no. AOT21999.1) amino acid sequences were compared with human YY1(GenBank accession no. NP_003394.1), *Xenopus tropicalis* YY1 (GenBank accession no. NP_001116880.1), Danio rerio YY1 (GenBank accession no. ADM86720.1), and Gallus YY1 (GenBank accession no. NP_001026381.1). * represents that the alignment sequence is consistent. REPO domain and four highly homologous zinc finger domains are highlighted in red and black, respectively. L: *Litopenaeus vannamei* YY1, G: Gallus YY1, H: human YY1, X: *Xenopus tropicalis* YY1, D: Danio rerio YY1. (**B**) At designated time points, the cells were collected and analyzed by immunoblotting with anti Flag-Tag mouse monoclonal antibody (Supplemental Materials). (**C**) Immunofluorescence assay (IFA) for identification of *Lv*YY1 proteins. At 48 h-post-infection (hpi), Sf-9 cell monolayers were fixed and stained with Flag mAb, followed by TRITC-coupled goat anti-mouse IgG. Fluorescence microscopy labeled nuclear DNA using DAPI. Infection was observed with inverted fluorescence microscope (magnification, 400×). (**D**) *Lv*YY1 bound to *ie1* promoter genome. Binding of purified *Lv*YY1 protein to *ie1* promoter DNA using the gel shift assay. *ie1* promoter DNA (200 ng), mutant *ie1* promoter (m*ie1*) and various concentrations (1 to 5 μg) of purified His-tagged recombinant *Lv*YY1 were mixed in binding buffer. The DNA-protein mixtures were subjected to 1% agarose gel electrophoresis to visualize changes of the DNA motility. (**E**) Binding of nuclear protein extraction from Sf-9 cells infected with *Lv*YY1 expressing baculovirus at 48 h to *ie1* promoter DNA. (**F**) Competition assay. *ie1* promoter, m*ie1* promoter and purified *Lv*YY1 protein were mixed in binding buffer. (**G**) Mutant *ie1* sequence. YY1, NF-κB, KLF, and TATA box sites in the promoter are indicated. m*ie1* and *ie1* sequence differ only in the YY1 binding sites.

Protein sequences alignment showed that *Lv*YY1 contains four highly conserved zinc fingers and recruit polycomb (REPO) domains like other proteins in the YY1 family (Figure 2A). To study the role of *Lv*YY1, amplified gene fragments were introduced into pOET5 to produce pOET5-*polh*-*Lv*YY1. pOET5 vector was used as control (Figure 1). Recombinant baculoviruses generated from these plasmids were used to infect Sf-9 cells. Western blot analysis identified *Lv*YY1 and revealed a band of 60 KDa (Figure 2B). *Lv*YY1 was also confirmed using immunofluorescent assay (IFA) with mouse anti-Flag mAb (Figure 2C).

To verify whether *Lv*YY1 is a common promoter enhancer, gel shift assays were used to examine the interaction of *Lv*YY1 with WSSV *ie1* promoter DNA. Incubation of recombinant *Lv*YY1 with *ie1* promoter DNA led to a retarded mobility pattern in a dose-dependent manner. *Lv*YY1 blocked *ie1* promoter DNA movement, but not the mutant (Figure 2D). The retarded band migration was also observed when *ie1* and mutant *ie1* were incubated together in binding buffer for competition assays (Figure 2F). To further confirm the interaction in BEVS, nuclear proteins were extracted from Sf-9 cells infected with *Lv*YY1 expressing baculovirus and tested in the same test. Retarded migration of *ie1* promoter was observed in the similar dose-dependent manner with baculovirus expressed *Lv*YY1 in Sf-9 cells (Figure 2E). These results suggested *Lv*YY1 is a specific promoter enhancer for WSSV *ie1* promoter, which is only valid via binding to special DNA sites.

### 3.2. LvYY1 Enhanced eGFP Expression Driven by WSSV ie1 Promoter

**Figure 3:**
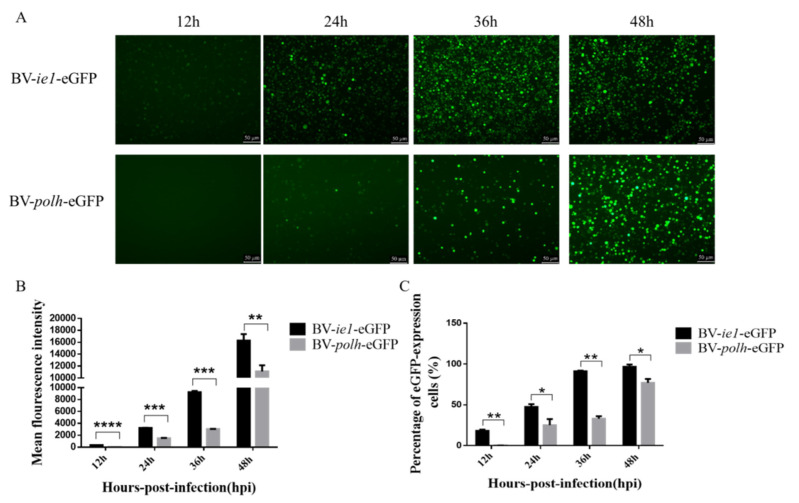
WSSV *ie1* promoter mediated stronger fluorescence intensity in Sf-9 cells than the conventional promoter. Sf-9 cells were infected with the same titer of recombinant baculovirus (BV-*ie1*-eGFP, BV-*polh*-eGFP). (**A**) Fluorescent microscopic analysis of infected Sf-9 cells at 12, 24, 36, and 48 hpi (magnification, 100×). (**B**) Sf-9 cells with infected recombinant baculovirus were collected for flow cytometry analysis. The mean fluorescence intensity detected using flow cytometry is quantified and displayed in the histogram. (**C**) The percentage of fluorescent cells was examined using flow cytometry. Data represented as the mean ± SD of three independent experiments. * *p* < 0.5, ** *p* < 0.01, *** *p* < 0.001, unpaired Student’s *t*-test.

To confirm the activity of WSSV *ie1* promoter, a plasmid containing WSSV *ie1* promoter was transfected into Sf-9 cells to test eGFP expression. *Polh* promoter was used as a control promoter (Figure 1). As shown in Figure 3A,B, *ie1* promoter mediated fluorescence intensity was much stronger than *polh* promoter. Moreover, the percentage of fluorescent cells driven by *ie1* is significantly higher than that driven by *polh* promoter (Figure 3C). These proved that *ie1* promoter has a strong promoter activity at the early phase.

**Figure 4:**
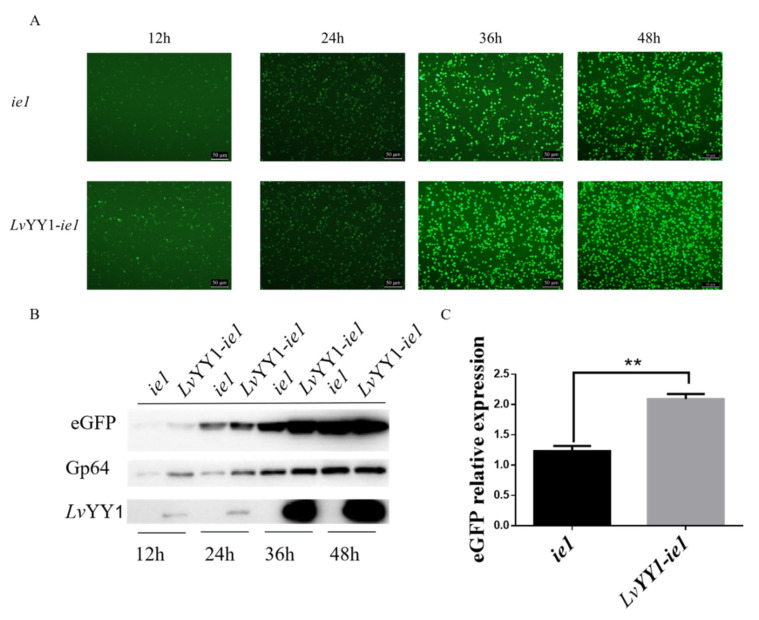
Comparative expression levels of eGFP based on interaction between WSSV *ie1* and *Lv*YY1. (**A**) Sf-9 cells were infected with the same titer of recombinant baculovirus. Fluorescence microscopy showed expression of eGFP under WSSV *ie1* promoter and *Lv*YY1 at different time points post-infection (magnification, 100×). (**B**) Total proteins were extracted at different time points post-infection, and subjected to Western blot analysis for eGFP and *Lv*YY1 expression. GP64 served as internal reference (Supplemental Materials). (**C**) The ratios of band intensity of eGFP to Gp64. Data represented as the mean ± SD of three independent experiments. ** *p* < 0.01, unpaired Student’s *t*-test.

Based on the above experiment, two expression cassettes were constructed, as shown in Figure 1, to evaluate the effect of *Lv*YY1 on the expression of eGFP reporter. Sf-9 cells were infected with the same titer of recombinant baculovirus. The emission of green fluorescence in Sf-9 cells was observed at different hpi, indicating the expression level of eGFP. Particularly, BV-*Lv*YY1-*ie1*-eGFP presented more and intensive fluorescence signals compared with BV-*ie1*-eGFP (Figure 4A). Then eGFP expression level was further confirmed using Western blot analysis of the total cellular extract with mouse anti-GFP mAb (Figure 4B). The expression level of eGFP was increased to 1.8-fold by *Lv*YY1 mediating WSSV *ie1* promoter (Figure 4C, *p* < 0.01).

### 3.3. LvYY1 Improved CSFV E2 Secretion under WSSV ie1 Promoter in Sf-9 Cells

**Figure 5:**
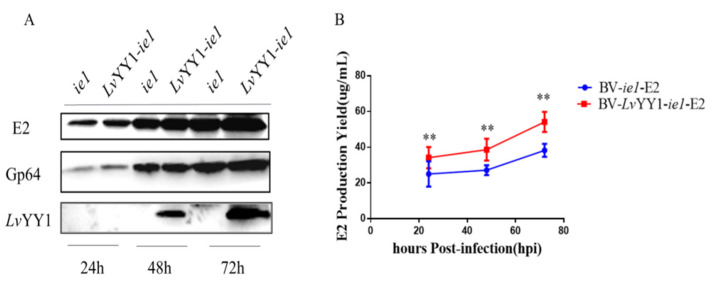
Comparative expression levels of CSFV E2 based on WSSV *ie1* and its enhancer (*Lv*YY1). (**A**) Western blot detection of *Lv*YY1 and E2 protein expression in Sf-9 insect cells infected with recombinant baculoviruses. BV-*ie1*-E2 or BV-*Lv*YY1-*ie1*-E2 infected Sf-9 cell lysates were harvested at different times after infection and used for analysis. E2 protein (55 kDa) was detected by mouse anti-E2 polyclonal antibody, and *Lv*YY1 protein (60 kDa) was analyzed by anti-Flag monoclonal antibody. Anti-Gp64 was included as an internal control (Supplemental Materials). (**B**) BV-*Lv*YY1-*ie1*-E2 and BV-*ie1*-E2 cultured in 6-well cell culture plates was compared with standard E2 protein solution (50 ug/mL). Results were shown as mean ± SD for three independent experiments. ** *p* < 0.01, unpaired Student’s *t*-test.

To evaluate the potential enhancement by the combination of *Lv*YY1 and WSSV *ie1* promoter in antigen expression, secreting CSFV E2 was used as the model in baculovirus. Baculovirus vectors were constructed to express E2 under WSSV *ie1* promoter either with *Lv*YY1 (pOET5-*Lv*YY1-*ie1*-E2) or without *Lv*YY1 (pOET5-*ie1*-E2). The expression of E2 in baculoviruses-infected cells was determined in Western blot (Figure 5A). E2 expression level in BV-*Lv*YY1-*ie1*-E2 infected Sf-9 cells was significantly higher than that of BV-*ie1*-E2 infected cells (Figure 5B, *p* < 0.01). Consistent with the result of Western blot analysis, E2 expression with BV-*Lv*YY1-*ie1*-E2 was up to 1.6-fold greater than BV-*ie1*-E2 infected Sf-9 cells (*p* < 0.01). These results indicated that *Lv*YY1 and WSSV *ie1* promoter could synergistically enhance the expression efficiency of CSFV E2.

### 3.4. LvYY1 Stimulated Active and Functional H5 Hemagglutinin Expression in Baculovirus

**Figure 6:**
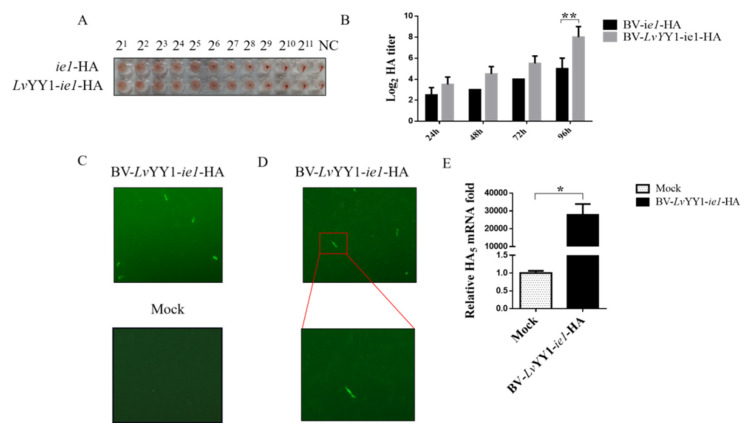
Comparative expression levels of H5 with WSSV *ie1* and its enhancer (*Lv*YY1). (**A**) Hemagglutination assays. Every 25 μL of baculovirus at a titer as loaded into the standard hemagglutination assay. (**B**) Hemagglutination titer of HA baculoviruses at different hpi. Results show mean ± SD for three independent experiments. ** *p* < 0.01, unpaired Student’s *t*-test. (**C**) BV-*Lv*YY1-*ie1*-HA was transduced in CEF cells for 12 h. Virus containing medium were removed, and the cells were incubated in fresh DMEM for an additional 48 h before being fixed for IFA. (**D**) Transduced CEF cells were magnified under 10 × 20 magnification. (**E**) H5 transduction efficiency was examined by qPCR using total RNA extracted from transduced CEFs 36 h post inoculation. Results show mean ± SD for three independent experiments. * *p* < 0.05, unpaired Student’s *t*-test.

To further verify the role of *Lv*YY1 as transcription enhancer in the production of other antigen types, such as baculovirus surface displayed antigen, AIV H5 expression was evaluated with *Lv*YY1 in baculovirus. Two recombinant baculoviruses expressing H5 were constructed and termed as pOET5-*ie1*-HA and pOET5-*Lv*YY1-*ie1*-HA. HA titers of baculoviruses were evaluated with the same amount of recombinant baculoviruses (Figure 6B) to indicate the active antigen expression. As shown in Figure 6B, supernatant of both baculoviruses showed hemagglutination activity. During the infection of 96 h, BV-*Lv*YY1-*ie1*-HA elicited higher levels of HA expression than did BV-*ie*1-HA. *Lv*YY1 displayed a higher mean hemagglutination titer up to 1:512 (BV-*Lv*YY1-*ie1*-HA), while those baculoviruses without *Lv*YY1 stimulation were at a mean titer of 1:64 (BV-*ie1*-HA) at 96 hpi (Figure 6A). HA yield of BV-*Lv*YY1-*ie1*-HA was higher than that of BV-*ie1*-HA at the same MOI and culture scale, indicating its advantage in functional HA production.

### 3.5. LvYY1-ie1 Baculovirus Mediated H5 Transduction in CEF Cells

To test the transduction potential of BV-*Lv*YY1-*ie1*-HA in target cells, CEF cells were incubated with BV-*Lv*YY1-*ie1*-HA in MOI of 100 for 12 h. Compared with mock cells, the number of CEF cells incubated with BV-*Lv*YY1-*ie1*-HA was greatly reduced, accompanied by cell shedding and lysis. Moreover, H5 specific fluorescent signals in IFA were detected in BV-*Lv*YY1-*ie1*-HA cells but not in the mock cells (Figure 6C), indicating the successful H5 transduction in CEFs via BV-*Lv*YY1-*ie1*-HA. Moreover, the transduction of BV-*Lv*YY1-*ie1*-HA was verified with qPCR (*p* < 0.05, Figure 6E). Significant HA transcription was detected in transduced CEF cells after the incubation with BV-*Lv*YY1-*ie1*-HA for 36 h, while the mock group showed negative detection. These results demonstrated that BV-*Lv*YY1-*ie1*-HA not only displays sufficient AIV HA protein on the viral envelope as antigens, but also acts as a vector to mediate transduced HA expression in CEF cells.

### 3.6. LvYY1-ie1 Baculovirus Presented Improved Protective Immunogenicity in Chicken against H5 AIVs

To explore the immunogenicity and efficacy of *Lv*YY1 against H5 AIVs, SPF chickens were immunized with either BV-*Lv*YY1-*ie1*-HA or BV-*ie1*-HA without adjuvant, as well as emulsified commercial inactivated vaccine and PBS controls individually. HA specific protective immune response was evaluated two weeks after each immunization.

**Figure 7:**
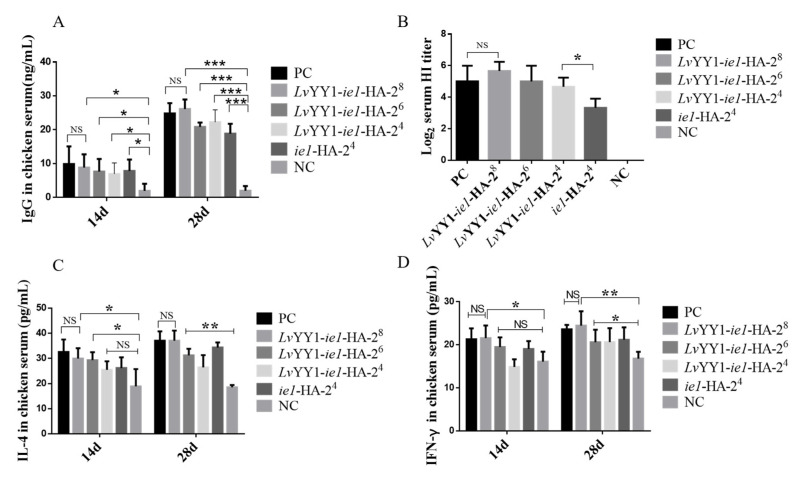
Efficient immunogenicity of BV-*Lv*YY1-*ie1*-HA in chickens. Two-week-old specific pathogen-free (SPF) chickens were randomly divided into 6 groups (A, B, C, D, E, and F, *n* = 5). Groups A to C were respectively immunized twice with different hemagglutination titers (2^8^, 2^6^, 2^4^) of 200 μL live baculoviruses containing HA antigen without adjuvant (BV-*Lv*YY1-*ie1*-HA). Group D was immunized with 200 μL 2^4^ hemagglutination titers of BV-*ie1*-HA. Group E was immunized with emulsified commercial inactivated vaccine as a positive control (PC). Group F was immunized with PBS as a negative control (NC). (**A**) Detection of HA-specific antibodies in chicken serum with ELISA. (**B**) Three serum samples were randomly collected on day 28 from each group for measuring the serum hemagglutination inhibition (HI) titer. (**C**) Serum IL-4 concentration following vaccination with recombinant baculovirus vaccines. (**D**) Serum IFN-γ concentration following vaccination with recombinant baculovirus vaccines. Results show mean ± SD for three independent experiments. * *p* < 0.05, ** *p* < 0.01, *** *p* < 0.001, unpaired Student’s *t*-test.

As shown in Figure 7A, all sera induced by different dosages of BV-*Lv*YY1-*ie1*-HA presented specific antibody response against H5 AIVs in a commercial AIV antibody test kit. Furthermore, the experimental group of 2^8^ hemagglutination titers was higher than commercial inactivated vaccine, even if was not emulsified. Moreover, at the same dosage of HA inoculated, chickens immunized with those baculoviruses under *Lv*YY1 mediated-*ie1* promoter developed higher antibody response than those under *ie1* promoter but without *Lv*YY1, which may be caused by the higher transducing efficiency in vivo with *Lv*YY1 activation after immunization.

Antiviral function of sera was tested in HI assays against H5 AIVs (Figure 7B). All sera showed positive HI activity, except for the negative control. Sera of chickens immunized with BV-*Lv*YY1-*ie1*-HA exhibited slightly enhanced HI activity compared to commercial inactivated vaccine. However, a significant difference was observed between chickens immunized with BV-*Lv*YY1-*ie1*-HA (2^4^) groups and BV-*ie1*-HA (2^4^) immunized chicken groups (*p* < 0.05).

Cytokine levels in immune sera can be used to measure humoral immune response. IL-4 is secreted by T-helper type 2 (Th2) cells, and IFN-γ are secreted by Th1 cells, which play important roles in regulating cellular (T cell) immune response. Thus, it is possible to determine whether the baculovirus constructs are eliciting a predominately Th1 or Th2 response by comparing the levels of cytokine production. The level of IL-4 increased significantly (*p* < 0.05) at 14 and 28 days post primary vaccination in the immunized groups compared to the negative control groups (NC; Figure 7C). The highest level of IL-4 were observed in BV-*Lv*YY1-*ie1*-HA (37.07 pg/mL) groups. The IFN-γ level of BV-*Lv*YY1-*ie1*-HA (24.43 pg/mL) was not significantly different from that of the positive control group (emulsified commercial inactivated vaccine, 23.59 pg/mL, Figure 7D), possibly due to the absence of adjuvant. This study provided evidence that the baculovirus constructs elicited a robust cellular immune response in immunized chickens.

## 4. Discussion

Vaccination is the principal means to prevent and control diseases. Subunit vaccines have many advantages over other vaccines including higher safety, better stability, and long-lasting immunization. The unique features of BEVS make them especially suitable for subunit vaccine antigen production. Although BEVS has been widely used for antigen production [37], this system is limited in terms of productivity. It is notable that novel approaches to improve yield are in urgent need to broaden BEVS application for industrial and scientific purposes. A wide range of strategies have been proposed for increasing productivity, including the modification of baculovirus promoters [30] and/or combined use of regulatory sequences [38,39,40].

The *ie1* promoter carried by WSSV has strong promoter activity throughout the baculovirus infection cycle [41]. In many studies, the heterologous *ie1* promoter is used as a powerful promoter for expressing foreign protein in BEVS [42]. For example, YuYang proved that the WSSV *ie1* promoter was the strongest promoter activity among the *polh* promoter, ETL promoter, mETL promoter, *ie1* promoter, and m*ie1* promoter. In our experiments, we also found that the WSSV *ie1* promoter can drive higher eGFP expression before 48 h (Figure 3). This finding also confirmed that the WSSV *ie1* promoter exerted a strong promoter activity at early phase. However, the efficiency of promoter is attributed to the regulatory sequences to a large extent. To the date, there have been no reports about the application of the regulatory sequence of the WSSV *ie1* promoter.

Yin Yang 1 (YY1) is widely expressed in many cell types, including mammalian and insect cells, and regulates transcription via interactions with associated cofactors [30]. It was found that *Lv*YY1 from shrimp could bind to the *ie1* promoter to activate WSSV viral transcription, but the effect of *Lv*YY1 on the expression of recombinant protein driven by *ie1* promoter in BEVS has not been evaluated. Our study firstly aimed to investigate their influence on gene expression in BEVS.

In the present study, we found that *Lv*YY1 could be heterogeneously expressed in Sf-9 cells and located in the nucleus. *Lv*YY1 protein size was identified to be 60 KDa, which was consistent with previous studies. It was proved that the ratio of *Lv*YY1 acidic amino acids was up to 20.6%, which may affect protein migration in SDS-PAGE [30]. Subsequently, a gel shift assay experiment, a classic technique to study the interaction between DNA binding proteins and their associated DNA binding sequences, was performed to verify the interaction between WSSV *ie1* promoter and *Lv*YY1 in BEVS. As shown in Figure 2D,E, *Lv*YY1 and *ie1* combination caused nucleic acid migration, and this effect was dose-dependent. It is the first time that the transcriptional element of *ie1* promoter has been found in BEVS.

To evaluate the protein expression based on the interaction between *Lv*YY1 and WSSV *ie1* promoter, two baculovirus constructs were developed using eGFP as reporter gene. As shown in Figure 4A,B *Lv*YY1 could significantly increase *ie1* promoter-mediated eGFP expression in Sf-9 cells, whereas the control vector could not. Therefore, it could be concluded that *Lv*YY1 effectively enhanced the *ie1* promoter-mediated gene in Sf-9 cells. These findings showed that *Lv*YY1 has great potential in BEVS to improve antigen expression level.

The system was firstly evaluated for secreting protein production in Sf-9 cells. CSFV E2 is widely used in vaccine production against CSF. At present, E2 subunit vaccine produced by insect cells has not been widely used due to limited production [43]. Many strategies have been applied to improve E2 expression, such as fusion with a certain signal peptide [44]. Furthermore, efforts have been made to select promoters, such as p10, a minimal *Drosophila melanogaster* Hsp70 promoter [45]. In this study, it is notable that BV-*Lv*YY1-*ie1*-E2 induced stronger antigen expression, potentially because it has the transcription enhancer *Lv*YY1 and WSSV *ie1* promoter. E2 antigen expression was 1.6-fold higher than that of the control (Figure 4A). Compared to the standard E2 protein solution, E2 yield was up to 53 μg/mL in 6-well cell culture plates (Figure 4B).

Moreover, as baculovirus is applicable to different vaccine types, *Lv*YY1 was also studied here about the improvement of surface displayed HA. HA is an important antigen of AIV and mediates the binding of virus to the host cell receptor and then boosts the entry of virus genome into the host [46], which is also the main target of vectored vaccines, as well as subunit vaccines. The production of traditional influenza inactivated vaccines using SPF embryo eggs requires high level biosafety equipment and facilities [47]. There are also studies using BEVS to express HA protein [48]. In this study, WSSV *ie1* promoter and *Lv*YY1 were used to drive H5 subtype expression in Sf-9 cells and improved its yield, as shown by the increase of eight times in the hemagglutination titer.

Results (eGFP, E2, HA) here confirmed that *Lv*YY1 is a promising candidate to enhance vaccine antigen production. Furthermore, the role of *Lv*YY1 in vaccine function regulation was studied as well. Given that *ie1* promoter can exert promoter activity in a variety of host cells [49], the activity of BV-*Lv*YY1-*ie1*-HA in gene transduction in CEF cells was evaluated to determine its potential as vector vaccines. Compared to mock cells, BV-*Lv*YY1-*ie1*-HA successfully transduced CEF cells, demonstrating that baculovirus displaying HA with *Lv*YY1-WSSV-*ie1* machinery sustains the transduction activity in CEFs as a promising vaccine delivery vector.

To further verify the advantages that *Lv*YY1 brings in vaccine development, immunogenicity of these baculovirus expressed antigens was studied in chickens. Although the experimental group was not emulsified, the high neutralizing antibody titer and increased IL-4 and IFN-γ levels suggest that *Lv*YY1 could improve transduced antigen expression in vivo. Moreover, interestingly, even though chickens were injected with the same amount of HA antigen, the group of BV-*Lv*YY1-*ie1*-HA was found to elicit higher antibody response than the BV-*ie1*-HA group. The improved efficacy of BV-*Lv*YY1-*ie1*-HA may result from the enhanced transduction efficiency in chickens based on *Lv*YY1 as the transcription enhancer. Taken together, these findings suggested that *Lv*YY1 had the dual functions in WSSV *ie1* driven baculovirus for both recombinant viral vector and subunit vaccines. Our studies provide an alternative choice for the efficient production of innovative human and animal vaccines with baculovirus.

In summary, WSSV *ie1* promoter was reported as a strong promoter in insect cells. For the first time, we found that *Lv*YY1 activates antigen expression in BEVS by binding to the *ie1* promoter. The combination of *ie1* and *Lv*YY1 can increase the expression of exogenous antigens (for different antigen systems) and improve transduction efficiency. These findings demonstrate the importance of *Lv*YY1 for baculovirus antigen expression, which is a promising vaccine strategy based on BEVS. Further efforts will be made to explore the performance of *Lv*YY1 to other vaccine types, including vector vaccines and human vaccines.

## 5. Conclusions

Heterologous *Lv*YY1 could be expressed in Sf-9 cells, and interaction with WSSV *ie1* promoter (not m*ie1*) led to nucleic acid migration; the effect was dose-dependent, indicating that *Lv*YY1 specifically activates the WSSV *ie1* promoter in BEVS. On this basis, different antigen expression types (eGFP, E2, HA) were used to verify the role of *Lv*YY1 as a transcription element in transcription efficiency. Increased yields of eGFP, E2, and HA confirmed that *Lv*YY1 promotes vaccine antigen production via transcription enhancement of the WSSV *ie1* promoter. Moreover, transduction experiment and immunization study in chicken were used to verify the advantage of *Lv*YY1. BV-*Lv*YY1-*ie1*-HA sustains the transduction activity in CEF cells. BV-*Lv*YY1-*ie1*-HA promoted stronger antibody responses than did the positive control. In addition, the former stimulated significantly higher production of IL-4 and IFN-γ than the latter, indicating that Th1 and Th2 immune responses might be activated, which suggests that *Lv*YY1 has dual functions as vectored vaccines and subunit vaccines. Therefore, *Lv*YY1, as a WSSV *ie1* promoter enhancer, provides an alternative method for the production of various vaccines.

## Figures and Tables

**Table 1:** Primers used in this study.

Primer	Oligonucleotides (from 5′to 3′)	Product Size/bp
YY1-Flag-F	ATGGCCTCCTCCGACTTCGTGACCG	1089
YY1-Flag-R	TTACTTATCGTCGTCATCCTTGTAATCGGAGGAGTAGATCACGAATT	
ie1-F	ACGCGTTTGGCGTTTTATTTTCTTG	518
ie1-R	GGGCTTGAGTGGAGAGAGAGAGCTA	
eGFP-F	CTCTCTCTCCACTCAAGCCCATGGTGAGCAAGGGCGAGGAG	720
eGFP-R	TTACTTGTACAGCTCGTCCATGCCGAGA	
E2-F	ATGATAAAAGTATTAAGAGGGCAGG	1068
E2-R	CTACACGTCCAGGTCAAACCAGTAT	
HA-F	ATGGAGAAAATAGTGCTTCTTCTTGC	1704
HA-R	TTAAATGCAAATTCTGCATTGTAAC	
HA-F1	GTCAAGAAAGGGGACTCAGCA	181
HA-R2	GCCCAGTCGCAAGGACTAAT	
β-actin-F	GAG AAATTGTGCGTGACATCA	152
β-actin-R	CCTGAACCTCTCATTGCCA	

## Supplementary Materials

Supplementary Materials are available online at https://www.mdpi.com/2076-393X/8/3/510/s1.
