# Supplementary figures and images for: LvYY1 Activates WSSV ie1 Promoter for Enhanced Vaccine Production and Efficacy
Source: Vaccines (Basel). 2020 Sep 8;8(3):510. doi: 10.3390/vaccines8030510 (PMC7563808; doi:10.3390/vaccines8030510)

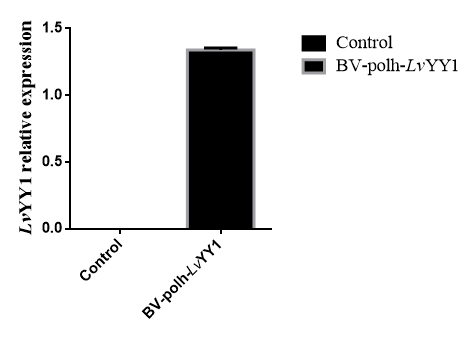

Supplement: Supplementary file 1 [file vaccines-08-00510-s001.zip › Supplemental Materials/figure.2B/Figure.2B intersity ratio.png]

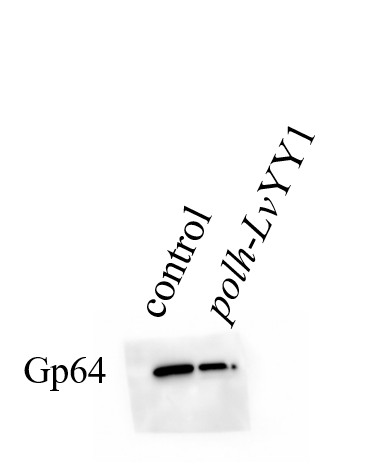

Supplement: Supplementary file 1 [file vaccines-08-00510-s001.zip › Supplemental Materials/figure.2B/Gp64.png]

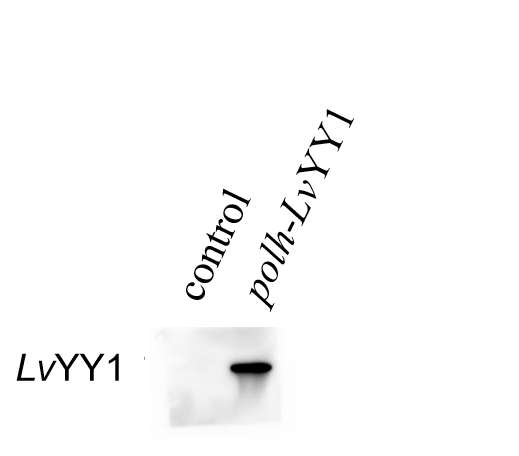

Supplement: Supplementary file 1 [file vaccines-08-00510-s001.zip › Supplemental Materials/figure.2B/polh-LvYY1.png]

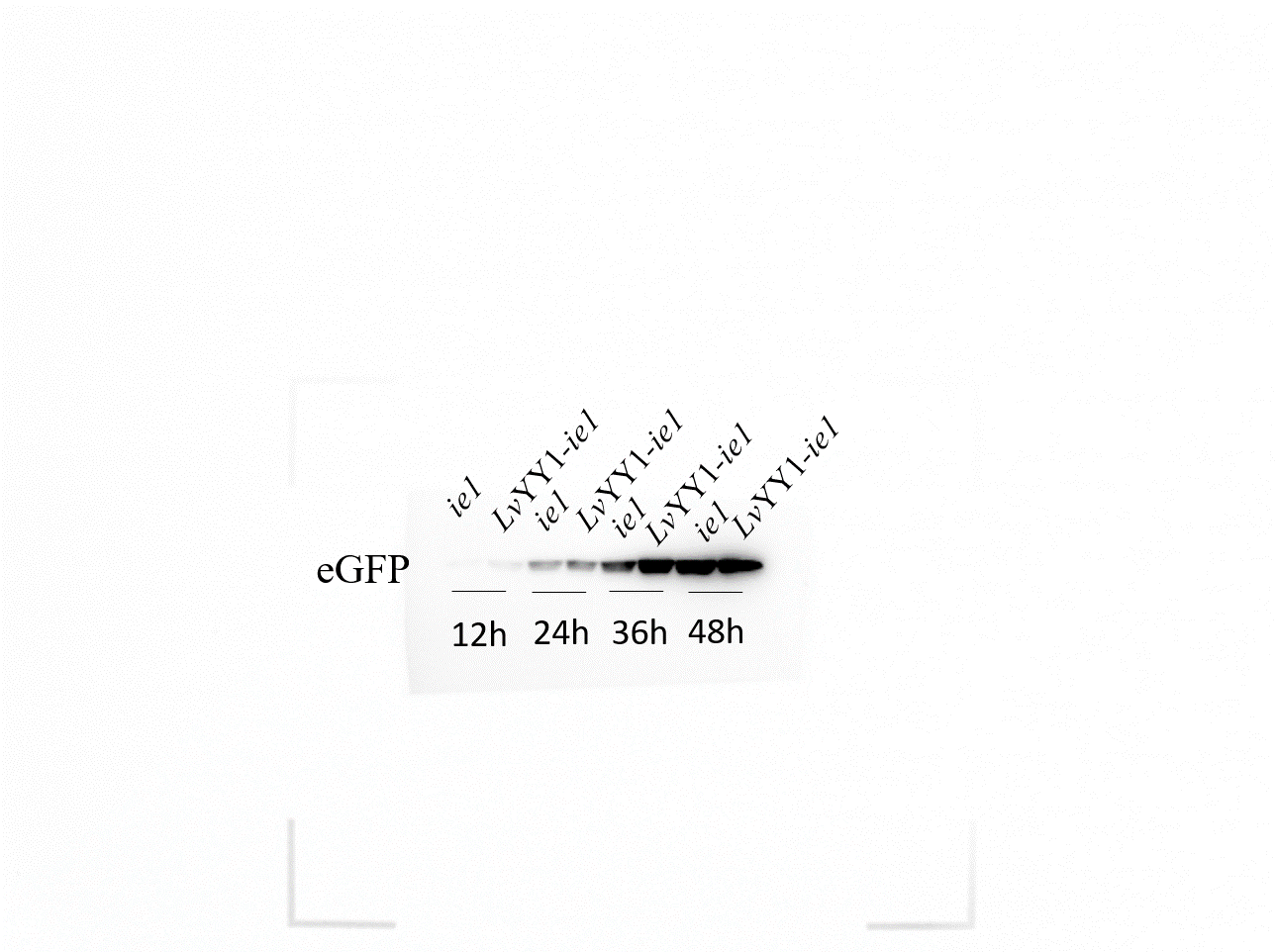

Supplement: Supplementary file 1 [file vaccines-08-00510-s001.zip › Supplemental Materials/figure.4B/eGFP.png]

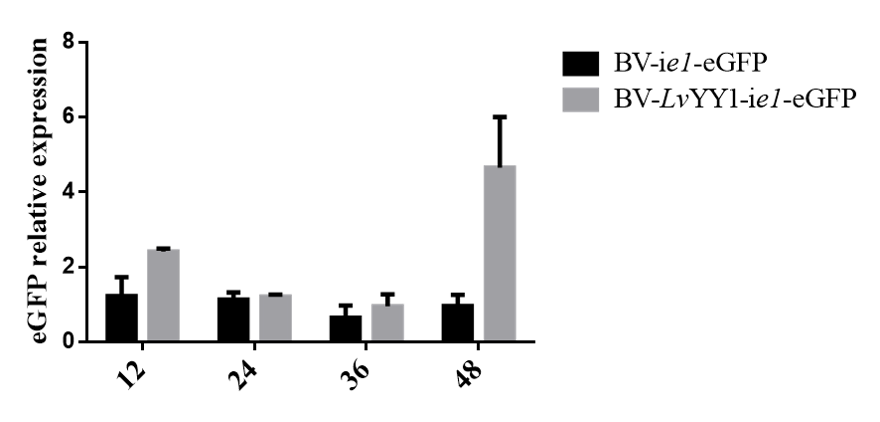

Supplement: Supplementary file 1 [file vaccines-08-00510-s001.zip › Supplemental Materials/figure.4B/Figure.4B intersity ratio.png]

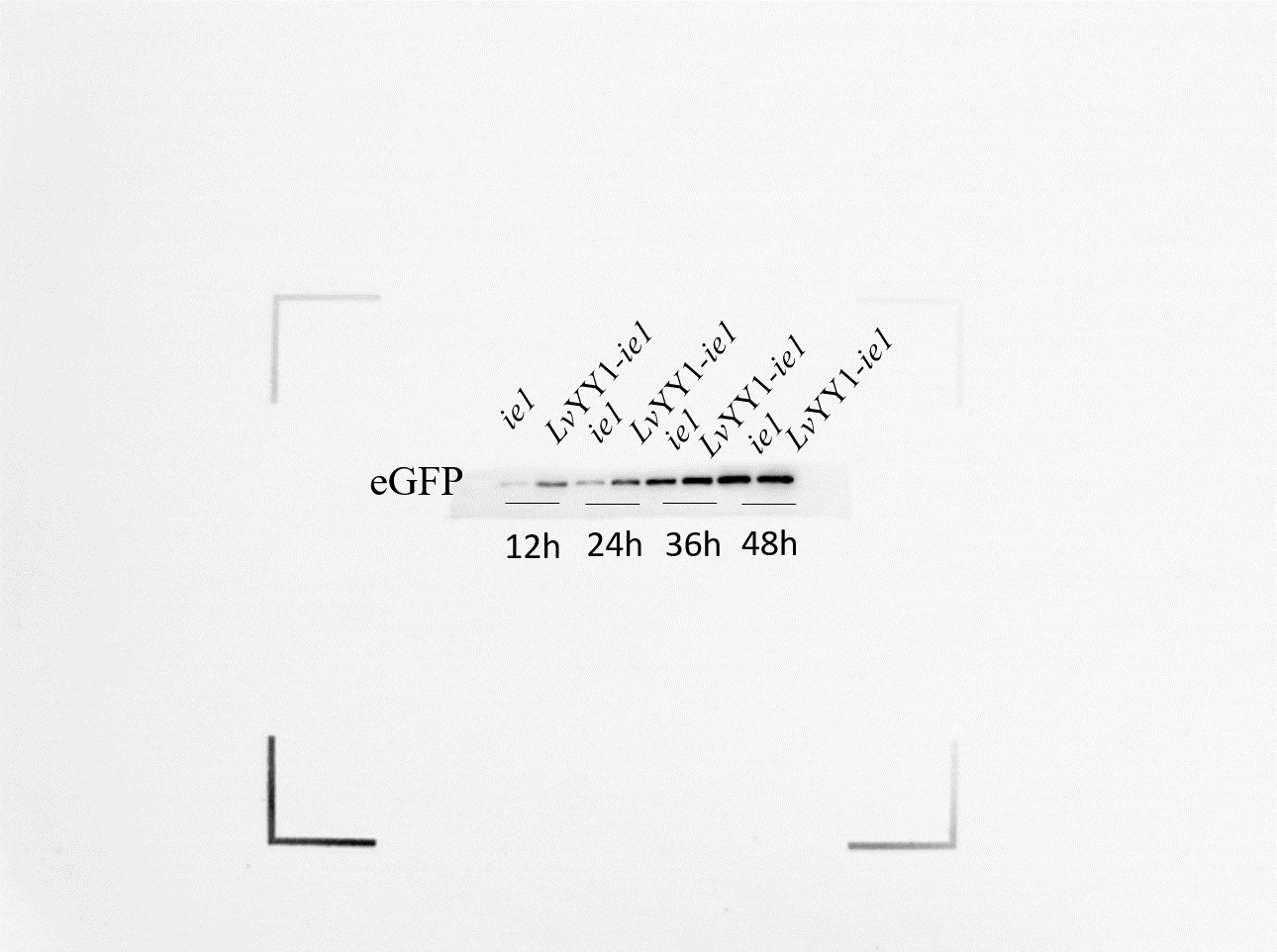

Supplement: Supplementary file 1 [file vaccines-08-00510-s001.zip › Supplemental Materials/figure.4B/Gp64.png]

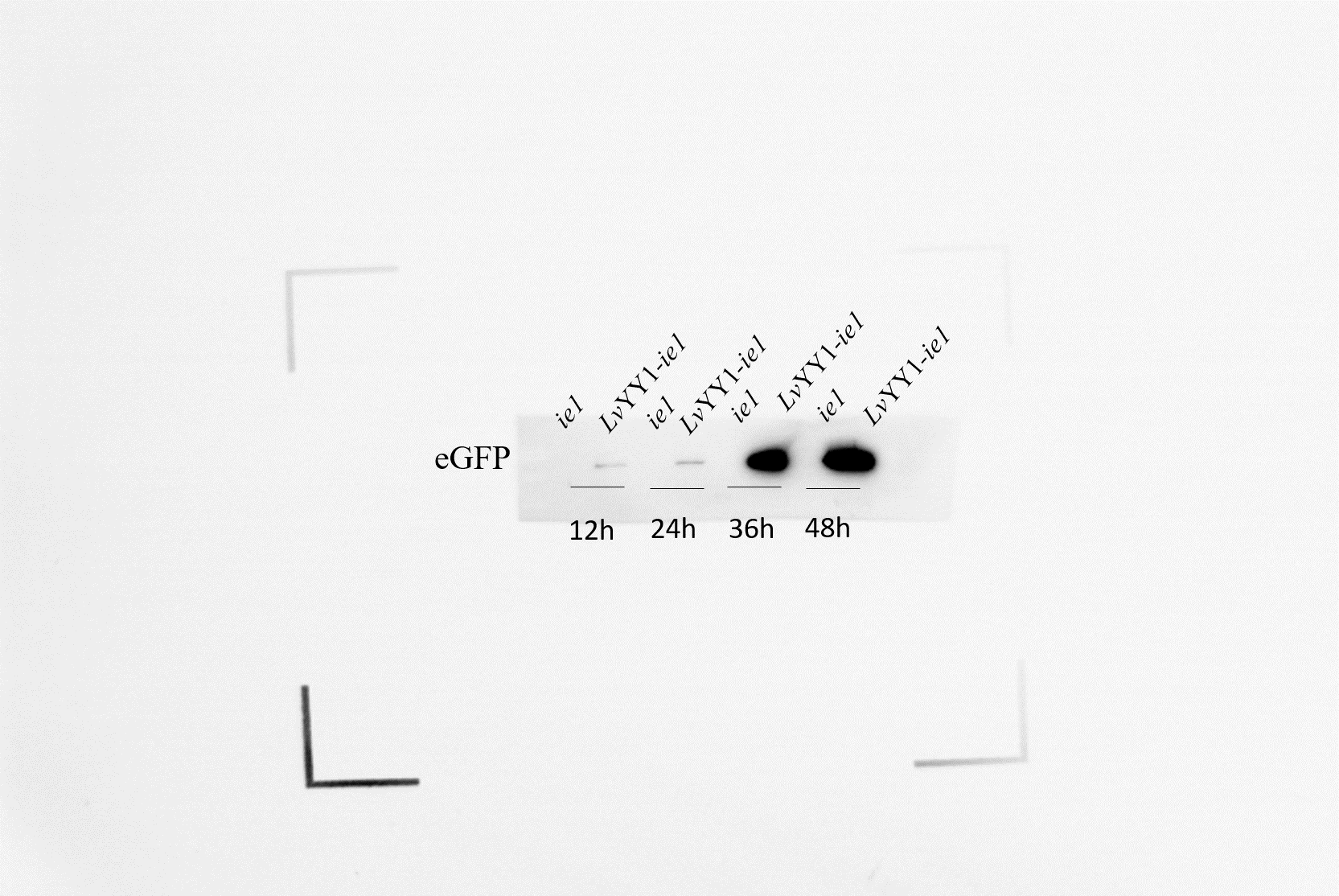

Supplement: Supplementary file 1 [file vaccines-08-00510-s001.zip › Supplemental Materials/figure.4B/LvYY1.png]

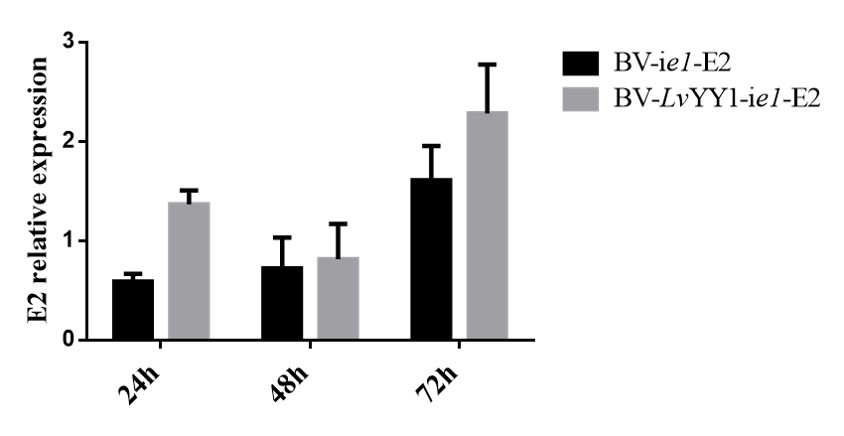

Supplement: Supplementary file 1 [file vaccines-08-00510-s001.zip › Supplemental Materials/figure.5A/E2 intersity ratio.jpg]

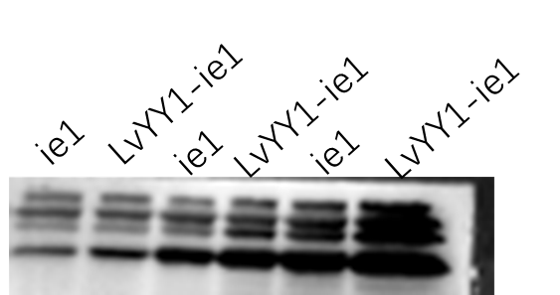

Supplement: Supplementary file 1 [file vaccines-08-00510-s001.zip › Supplemental Materials/figure.5A/E2.png]

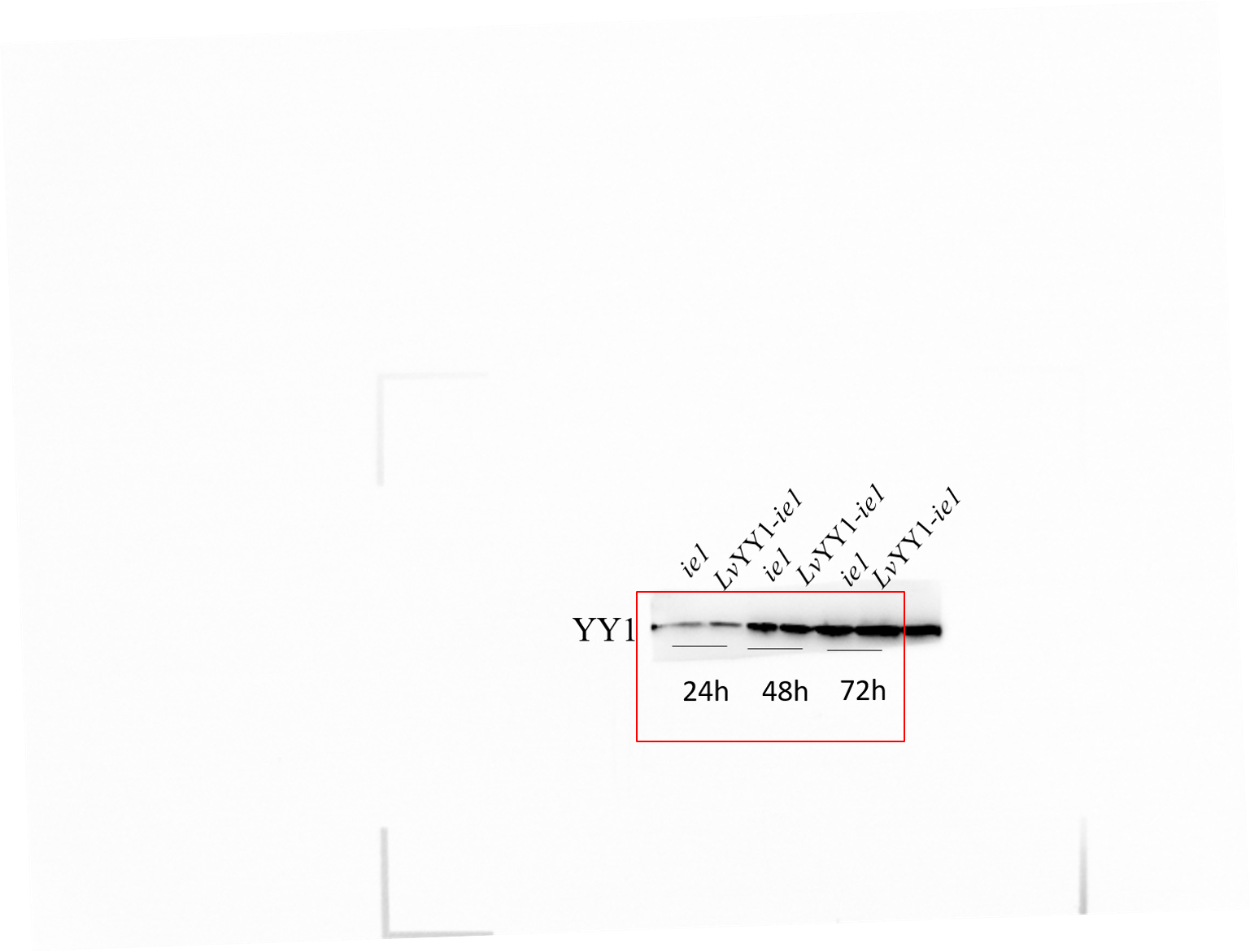

Supplement: Supplementary file 1 [file vaccines-08-00510-s001.zip › Supplemental Materials/figure.5A/Gp64.png]

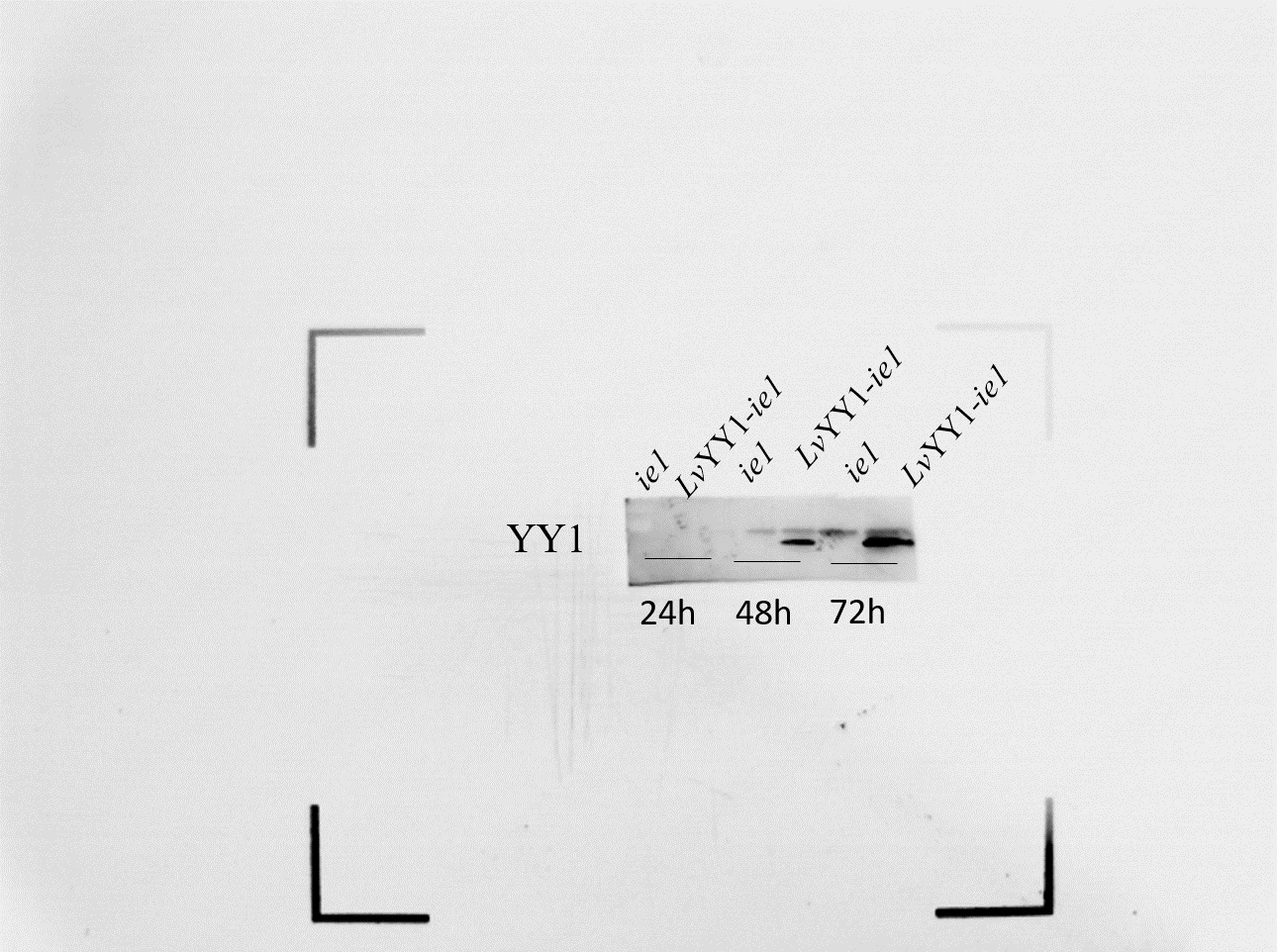

Supplement: Supplementary file 1 [file vaccines-08-00510-s001.zip › Supplemental Materials/figure.5A/LvYY1.png]
